# Lysosomotropic Properties of Weakly Basic Anticancer Agents Promote Cancer Cell Selectivity *In Vitro*

**DOI:** 10.1371/journal.pone.0049366

**Published:** 2012-11-07

**Authors:** Rosemary A. Ndolo, Yepeng Luan, Shaofeng Duan, M. Laird Forrest, Jeffrey P. Krise

**Affiliations:** 1 Department of Pharmaceutical Chemistry, University of Kansas, Lawrence, Kansas, United States of America; 2 Institute of Medicinal Chemistry, Shandong University, Jinan, Shandong, China; Enzo Life Sciences, Inc., United States of America

## Abstract

Drug distribution in cells is a fundamentally important, yet often overlooked, variable in drug efficacy. Many weakly basic anticancer agents accumulate extensively in the acidic lysosomes of normal cells through ion trapping. Lysosomal trapping reduces the activity of anticancer drugs, since anticancer drug targets are often localized in the cell cytosol or nucleus. Some cancer cells have defective acidification of lysosomes, which causes a redistribution of trapped drugs from the lysosomes to the cytosol. We have previously established that such differences in drug localization between normal and cancer cells can contribute to the apparent selectivity of weakly basic drugs to cancer cells *in vitro*. In this work, we tested whether this intracellular distribution-based drug selectivity could be optimized based on the acid dissociation constant (pKa) of the drug, which is one of the determinants of lysosomal sequestration capacity. We synthesized seven weakly basic structural analogs of the Hsp90 inhibitor geldanamycin (GDA) with pKa values ranging from 5 to 12. The selectivity of each analog was expressed by taking ratios of anti-proliferative IC_50_ values of the inhibitors in normal fibroblasts to the IC_50_ values in human leukemic HL-60 cells. Similar selectivity assessments were performed in a pair of cancer cell lines that differed in lysosomal pH as a result of siRNA-mediated alteration of vacuolar proton ATPase subunit expression. Optimal selectivity was observed for analogs with pKa values near 8. Similar trends were observed with commercial anticancer agents with varying weakly basic pKa values. These evaluations advance our understanding of how weakly basic properties can be optimized to achieve maximum anticancer drug selectivity towards cancer cells with defective lysosomal acidification *in vitro*. Additional *in vivo* studies are needed to examine the utility of this approach for enhancing selectivity.

## Introduction

Many anti-cancer drugs suffer from poor selectivity to target cells, leading to a high degree of toxicity and potentially life-threatening side effects; therefore, chemotherapy is often prematurely stopped, reducing the chances of a drug achieving its full potential. Anti-cancer drugs typically possess some degree of intrinsic selectivity towards cancer cells due to biochemical and/or metabolic differences between normal and transformed cells [1]–[3]. The intrinsic selectivity of an anticancer agent can be further enhanced using a variety of drug delivery approaches based on Ehrlich's proposed “magic bullet” [4], [5]. All of these targeting strategies share a common requirement, which is that the active cytotoxic agent must accumulate to a greater extent in or around transformed cells relative to normal cells. Although numerous creative strategies have been examined, such as prodrug strategies and antibody-drug conjugates, their therapeutic usefulness has been somewhat limited [6].

The intracellular distribution of a drug is a fundamentally important consideration for drug efficacy. Mammalian cells are extensively compartmentalized, and both drugs and their targets can have specific and discrete intracellular localization patterns. Accordingly, for the intended therapeutic effect to occur, the intracellular site of drug localization must be the same, to a certain degree, as that of the drug target. We and others have previously shown that physicochemical properties of drugs can predictably influence their intracellular localization pattern [7]–[10]. In addition, we have shown that a single drug can have substantially different intracellular localization and trafficking patterns in different cell types [11], [12].

The purposeful targeting of anticancer drugs to intracellular compartments in cancer cells represents an emerging area of exploration [13], [14]. For example, mitochondria have a negative membrane potential associated with their inner membrane, which has been shown to drive the accumulation of drugs with delocalized cationic charge [15]. In this regard, derivatives of Hsp90 inhibitors have been developed to exploit this finding and therefore selectively target a mitochondrial form of Hsp90 [16].

Alternatively, many weakly basic drugs with localized cationic charges have been shown to be extensively sequestered in acidic lysosomes of cells through an ion trapping mechanism, and the properties of the cell and drug that influence this have been reviewed [17]. Briefly, the pH of the lysosomes, relative to the pH of the cytosol, is one of the key physiological parameters that dictates the predicted degree of lysosomal accumulation [12], [17]. The greater the lysosome-to-cytosol pH gradient, the greater the extent of lysosomal sequestration is. For many amines, lysosomal trapping has been shown to be quite extensive, and is thought to approximate the total cellular accumulation [18]. Since drug targets are seldom localized in lysosomes, the extensive trapping of weak bases in this compartment can greatly reduce target interactions, thereby reducing drug activity.

Interestingly, we and others have shown that several cancer cell types have a lysosomal acidification defect [19]–[22]. In some instances, the lysosomal pH of cancer cells has been reported to be 2 pH units higher than the lysosomal pH of normal cells [22]. This elevation in lysosomal pH is predicted to have a profound impact on the intracellular distribution of weakly basic amines that are substrates for ion trapping in lysosomes (lysosomotropic agents). Specifically, the concentration of such drugs will decrease in the lysosomes of such cells and concomitantly increase in the cytosol as well as in other extralysosomal compartments. We propose that this difference in drug distribution behavior will facilitate an enhancement in drug activity in cancer cells relative to normal cells.

We have previously established the basis for this intracellular distribution-based drug (IDB) targeting platform using both *in vitro* and *in vivo* approaches [12], [23], [24]. Using cells grown in culture, we quantitatively measured the lysosome-to-cytosol concentration ratios of drugs in cells with low or elevated lysosomal pH. Weakly basic anticancer drugs were shown to redistribute from the lysosomes to the cytosol when lysosomal pH was elevated. This intracellular redistribution of drugs resulted in more pronounced cytotoxicity toward cells with elevated lysosomal pH. Importantly, such changes in intracellular distribution and toxicity were not observed for anticancer drugs without lysosomotropic properties. More recently, we established the basis for the IDB targeting platform in mice by evaluating the degree of drug-induced toxicity in mice with low or experimentally elevated lysosomal pH [24].

In this report we specifically questioned whether the lysosomotropic potential of a cancer drug correlated with the degree of IDB selectivity. This is a very important consideration that could guide the rational design of new anticancer agents with properties that optimize lysosomotropism and therefore IDB selectivity, or the modification of existing drugs to impart these properties.

## Results

### Selection and synthesis of analogs of geldanamycin with a range of weakly basic pKa values

We have previously shown that weakly basic anticancer drugs with lysosomotropic properties exhibit greater selectivity toward cancer cells than do their non-lysosomotropic counterparts [12]. In the present work, we tested the prediction that the lysosomotropic potential of weakly basic drugs correlates with their degree of differential selectivity toward cancer cells.

In addressing this question, we considered the drug-associated factors that are known to contribute to lysosomotropism. All substrates for ion trapping-based lysosomotropism are weakly basic, but not all weakly basic molecules are lysosomotropic. In his commentary on the subject, de Duve proposed that two independent drug-associated properties influence the lysosomotropic potential of a given drug. The first parameter is termed alpha and represents the lysosomal membrane permeability ratio for the drug in its ionized versus unionized forms. We have previously experimentally estimated alpha for a number of compounds by measuring octanol/buffer partition coefficients as a function of pH and correlated these values with lysosomal sequestration [7]. The second important drug-associated parameter is the pKa of the conjugate acid of the base. We have previously experimentally established how pKa of drugs influence lysosomal sequestration in cultured cells [8].

In this work, we chose to evaluate the influence of pKa on selectivity using inhibitors of the 0 kD heat shock protein (Hsp90) that systematically varied in their weak base pKa. We chose to modify the 17-position of the Hsp90 inhibitor GDA to create derivatives with varied pKa since previous reports had shown that modifications to this position did not alter activity [25].

To help guide our selection of derivatives with varying pKa, we considered a previously described theoretical relationship between weak base pKa and alpha on the lysosome-to-extracellular concentration ratio [17], which is given by the following expression.
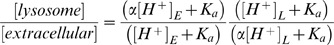



**Figure 1:**
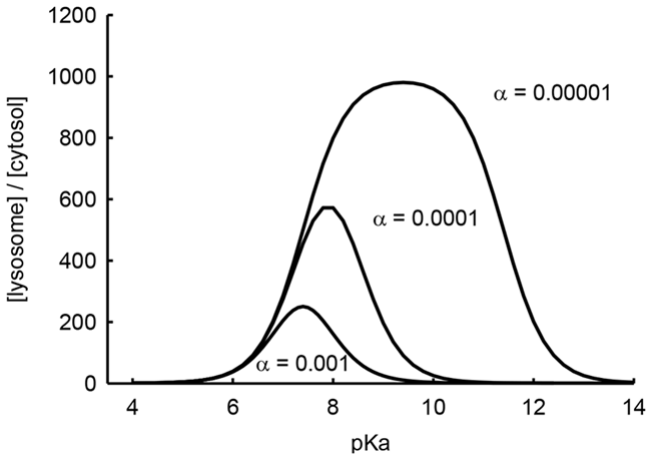
Theoretical plot of lysosomal sequestration as a function of weak base pKa and alpha (α). The equation used for these simulations can be found in the text. The pH of lysosomes and extracellular space were set at 4.4 and 7.4, respectively. The alpha parameter was varied as indicated.

The conjugate acid of weak base dissociation constant is denoted as K_a_, and [H^+^] denotes the proton concentrations (subscript E represents extracellular and subscript L represents lysosomal). The term alpha (α) denotes the ratio of lysosomal membrane permeabilities for the ionized base divided by that of the un-ionized species [26]. Using this equation, we calculated the theoretical lysosome-to-extracellular concentration ratio of a series of drugs with pKa values ranging from 4 to 14. For the calculations, we used lysosomal and extracellular pH values of 4.4 and 7.4, respectively, which are typical values for normal cells [27], and an alpha value of 0.01, which we had previously measured for 17-DMAG, a weakly basic derivative of GDA [12]. To demonstrate the potential influence of alpha on lysosomal sequestration, we have included plots in which this parameter is varied. Plotting the predicted lysosome-to-extracellular concentration ratios against pKa gave a bell-shaped curve, whereby the maximum degree of lysosomal sequestration and the pKa at which this occurred were sensitive to the magnitude of the alpha parameter (see Figure 1). Based on these simulations, we postulated that anticancer agents that vary in pKa from approximately 5 to 12 would adequately characterize the influence of lysosomal sequestration on tumor selectivity.

**Figure 2:**
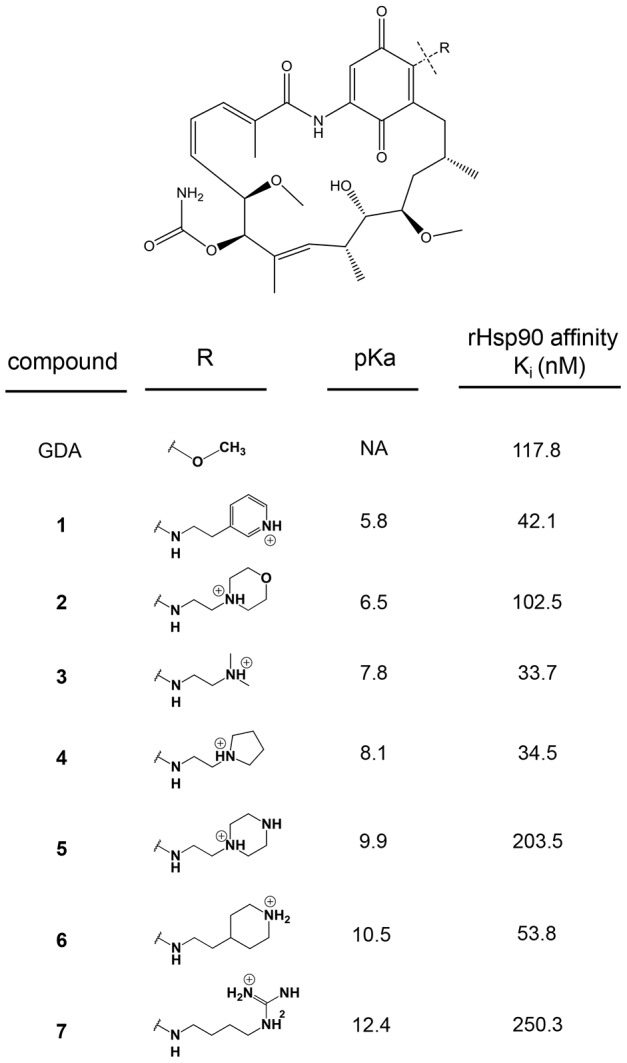
Structures and properties of geldanamycin derivatives. The seven derivatives with different weak base modifications at the 17-position of GDA are shown. The pKa values for derivatives 1, 2, 3, 5 and 6 was measured experimentally using proton NMR (n = 1). The pKa for compound 4 was measured experimentally in an earlier manuscript (see Results). The pKa for compound 7 was estimated using software (see Materials and Methods). The binding affinity for each of the inhibitors with rHsp90 is shown (n = 2), and was based on a previously established fluorescence polarization assay (see Materials and Methods).

We therefore synthesized a number of weakly basic analogs of GDA in an attempt to develop of small set of derivatives that had pKa values spanning this range. The GDA derivatives were synthesized as described in Materials and Methods and characterized by ^1^H-NMR (see Text S1). The pKa values for analogs 1, 2, 3, 5 and 6 (see Figure 2 for chemical structures) were measured at 37°C using ^1^H NMR. To determine the pKa using this method, the chemical shifts of protons alpha to the ionizable amine were plotted against pH, yielding sigmoidal curves (Figure S1) from which pKa was derived. For compounds 4 and 7, pKa could not be measured by ^1^H NMR due to either limited solubility in D_2_O and/or poorly resolved proton peaks. Therefore, we report a previously measured pKa value for compound 4 that was procured using an alternative approach [12], and we report a calculated value for compound 7 (see Materials and Methods). Compound 5 had two pKa values of 9.9 and 4.2. Only the higher pKa value is reported in Figure 2 because the second is too low to theoretically contribute to ion trapping in lysosomes. Collectively, the pKa values for each of the derivatives ranged from 5.8 to 12.4 (Figure 2).

To appropriately test the influence of weak base pKa on selectivity, all derivatives of GDA should retain some capacity to bind Hsp90 and act as inhibitors. To assess this, we evaluated the binding affinity of each of the derivatives to recombinant Hsp90 and compared the binding affinity with that of unmodified GDA. Binding affinities were estimated using a fluorescence polarization assay that measures the ability of test compounds to competitively displace FITC-conjugated GDA from Hsp90. The polarization (mP) values were converted to percent of control (no drug) and plotted against the concentration of test compounds, yielding sigmoidal curves from which the IC_50_ value was determined through curve fitting (Figure S2). The K_i_ values were calculated from IC_50_ values as previously described [28] using an online calculator (freely available at http://sw16.im.med.umich.edu/software/calc_ki/). The calculated IC_50_ for underivatized GDA was 230 nM, which is similar to previously reported values obtained using this assay [29]. A comparison of Ki values for GDA and its derivatives suggests that they all retain some capacity to bind Hsp90 and are therefore suitable for the evaluations described here (Figure 2).

### Assessment of intracellular distribution-based selectivity

**Figure 3:**
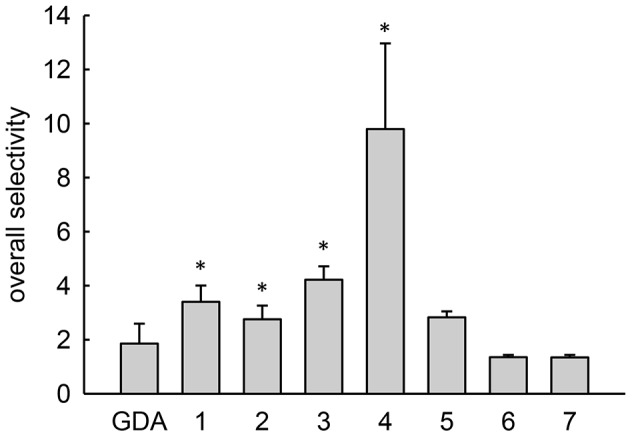
Overall selectivity comparisons for geldanamycin and its derivatives with varying pKa. Overall selectivity represents the IC_50_ for a derivative in normal human fibroblasts divided by the value obtained in HL60 human leukemic cells. Bars represent mean±s.d. (n = 3); *, *p*<0.05 compared to GDA.

To quantitatively express selectivity, we comparatively evaluated antiproliferative IC_50_ values in normal and cancer cells that had low and elevated lysosomal pH, respectively. For these evaluations, we utilized the HL60 human leukemic cell line, which we have previously shown to have an elevated lysosomal pH [22]. For the non-transformed cells, we utilized normal human foreskin fibroblasts that had previously been determined to have a lysosomal pH of 4.4 [22]. The cytotoxicity curves and IC_50_ values obtained from them are shown in Figure S3. By dividing the IC_50_ determined in normal cells by the IC_50_ in cancer cells, we obtained the overall selectivity (Figure 3). The non-lysosomotropic inhibitor GDA had an overall selectivity near two, which indicates that GDA was nearly twice as active in the leukemic cell line as it was in the normal cells. Interestingly, weakly basic inhibitors with pKa values near 8 had several-fold greater activity in the leukemic cells with elevated lysosomal pH compared to normal cells. Analogs of GDA that had pKa values far above or below 8 had no apparent increase in selectivity towards the cancer cells compared to underivatized GDA. On the whole, the influence of pKa on selectivity tended to yield a bell-shaped distribution that was similar to the theoretical plot correlating pKa with lysosomotropism (Figure 1).

Selectivity is multifactorial, and any successful cancer drug should exhibit greater activity toward cancer cells relative to normal cells through mechanisms that are unrelated to changes in intracellular distribution behavior that are evaluated in this manuscript [2], [3]. To directly the examine the selectivity that can be specifically attributed to changes in intracellular distribution behavior between normal and transformed cells, we considered it imperative to compare the antiproliferative activity of a given drug in two cell lines that are otherwise identical but have low (normal) and elevated (cancer-like) lysosomal pH. This approach would be most desirable because any inter-cell variability in factors that can influence drug activity (e.g., expression of drug efflux transporters) would be mitigated.

**Figure 4:**
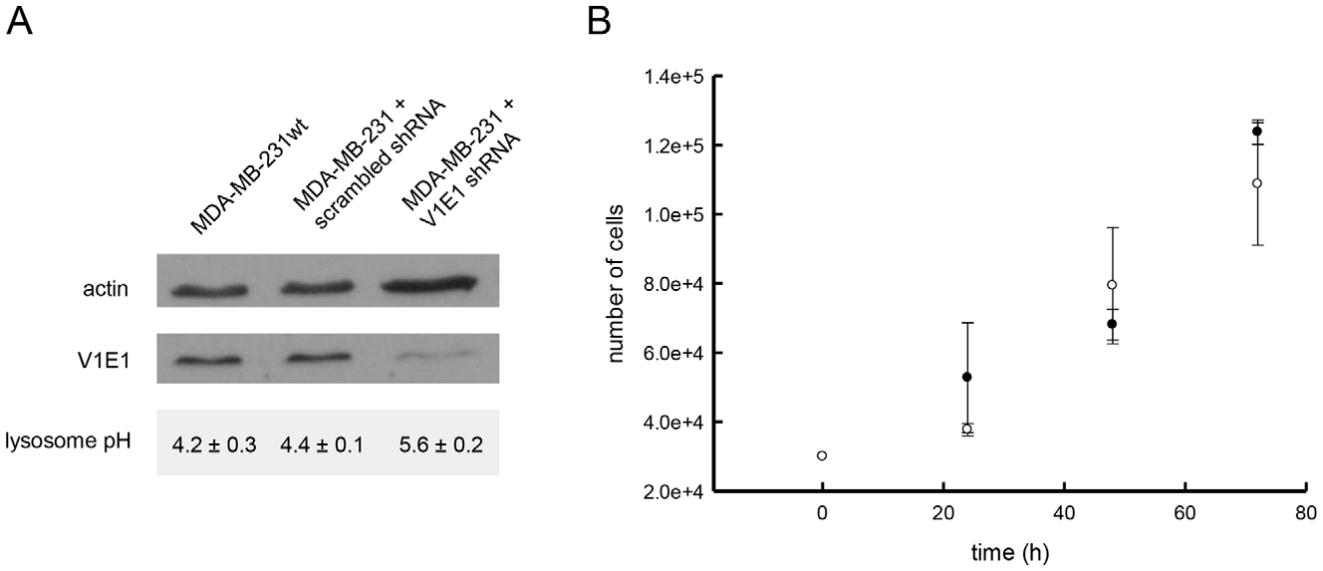
Characterization of MDA-MB-231 cells with reduced V-H^+^-ATPase subunit V1E1 expression and increased lysosomal pH. A. Western blot analysis of the V1E1 subunit expression is shown along with the actin loading control. Experimentally determined lysosomal pH values in untreated, scrambled shRNA- and V1E1 shRNA-treated cells is shown (values represent the mean±s.d., n = 3) B. V-ATPase subunit knockdown does not alter the growth rate of MDA-MB 231 cells. The filled circles represent cells treated with the scrambled shRNA vector and the open circles represent cells treated with the V-ATPase V1E1 shRNA. Cells were plated at a density of 3×10^5^ cells/well on a 6-well plate and were trypsinized, counted and replated every 24 hours for 3 days (data points represent the mean±s.d, n = 3).

Although many cancer cell lines have been shown to have defective acidification of lysosomes, we found that a number of them maintain normally acidified lysosomes. For example, the MDA-MB-231 breast adenocarcinoma cell line maintains a lysosomal pH of 4.2, similar to the lysosomal pH of most normal cells [27]. Using this cell line, we explored an siRNA approach to transiently elevate lysosomal pH. Lysosomal pH is regulated by the vacuolar proton ATPase (V-H^+^-ATPase) [30], and Lu and coworkers have previously shown that reducing the expression of a subunit of this enzyme, using siRNA, yielded cells with altered V-ATPase proton pumping activity, with no effect on cell proliferation [31]. We utilized a similar technique to create MDA-MB-231 cells with elevated lysosomal pH. Specifically, we targeted the V1E1 subunit of V-ATPase for knockdown using lentiviral vector shRNA. The lentiviral-mediated transfer was chosen because this approach allows for efficient, high-throughput and stable knockdown of target proteins [30]. MDA-MB-231 cells that were treated with lentiviral particles containing non-target (scrambled) sequence shRNA retained low lysosomal pH and served as an important control for any non-specific effects of the lentiviral infection on cell viability. Knockdown of V1E1 expression in cells receiving target viral particles was confirmed using Western blot analysis (Figure 4A). Importantly, knockdown of the V1E1 subunit led to no discernible effects on the growth of cells over the time course of the IC_50_ evaluations (Figure 4B). Experimental evaluation of lysosomal pH after knocking down the V-H^+^-ATPase subunit showed that lysosomal pH was significantly elevated from pH 4.4 to pH 5.8 (Figure 4A).

**Figure 5:**
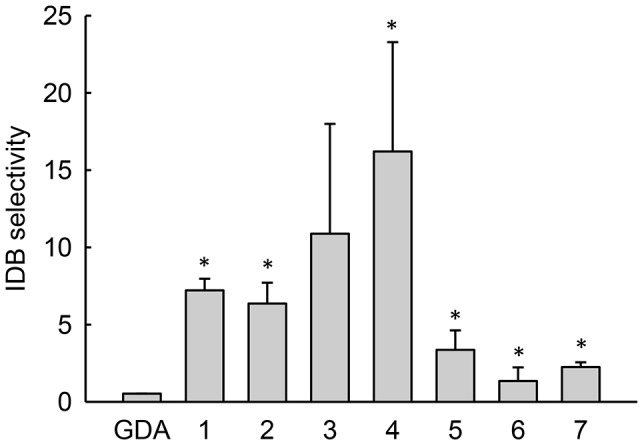
Intracellular distribution-based drug selectivity comparisons for geldanamycin and its derivatives with varying pKa. IDB selectivity is defined as the IC_50_ in MD-MB-231 cells treated with scrambled shRNA, which have low lysosomal pH, divided by the value obtained from the same cell line treated with shRNA against the V1E1 subunit of the vacuolar proton ATPase, which have elevated lysosomal pH. Bars represent mean±s.d. (n = 3); *, *p*<0.05 compared to GDA.

We subsequently evaluated the IC_50_ of the Hsp90 inhibitors in both cell types (Figure S4) and calculated selectivity as IC_50_ in MDA-MB-231 cells with low lysosomal pH divided by IC_50_ in MDA-MB-231 cells with elevated lysosomal pH. Consistent with our expectations, the activity of non-lysosomotropic GDA was not influenced by the lysosomal pH status of the cell and therefore had no apparent IDB selectivity (i.e., IDB selectivity value near one, see Figure 5). The trends in IDB selectivity as a function of Hsp90 inhibitor pKa followed a similar bell-shaped profile as did the overall selectivity results shown in Figure 3. Moreover, comparisons of the magnitude of the IDB with overall selectivity suggest that IDB selectivity is a major contributing factor to the overall selectivity of these agents.

Our previous results are consistent with the notion that Hsp90 inhibitors with optimal lysosomotropic properties (i.e., pKa values near 8) will possess the greatest degree of IDB selectivity toward transformed cells. To further evaluate this, we evaluated the IDB selectivity of additional classes of anticancer agents that do or do not possess lysosomotropic properties.

**Figure 6:**
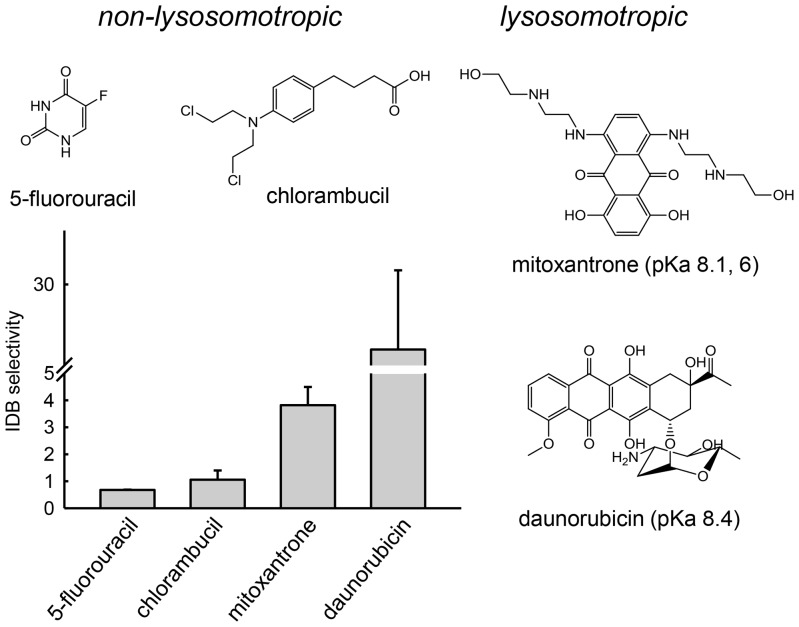
Only lysosomotropic anticancer agents possess significant intracellular distribution-based drug selectivity. Mitoxantrone and daunorubicin are weakly basic with pKa values near 8 and have significant IDB selectivity. Non-lysosomotropic anticancer agents 5-fluorouracil and chlorambucil have IDB selectivity values near 1, which demonstrates that their activity is not influenced by lysosomal pH. Bars represent mean±s.d. (n = 3).

Mitoxantrone and daunorubicin are weakly basic anticancer agents that have nuclear targets and pKa values near 8 [32], which should promote lysosomotropism and IDB selectivity. Alternatively, the antimetabolite 5-fluorouracil is weakly acidic, with a pKa of 8 [33], and is thus not considered a substrate for ion trapping in acidic lysosomes. 5-Fluorouracil should therefore neither have intracellular distribution nor activity that is influenced by the lysosomal pH status of a cell. Similarly, anticancer agents that are zwitterionic, with both weakly acidic and weakly basic groups, such as chlorambucil [34], are not lysosomotropic and should not exhibit IDB selectivity. Consistent with our reasoning, the non-lysosomotropic anticancer agents 5-fluorouracil and chlorambucil had IDB selectivity values near one, similar to GDA (Figure 6). Alternatively, the lysosomotropic agents mitoxantrone and daunorubicin both showed significant but different degrees of IDB selectivity (Figure 6). The cytotoxicity curves for these evaluations are shown in Figure S5.

## Discussion

In this study we have, for the first time, systematically investigated how the pKa of weakly basic anticancer agents influences the degree of IDB selectivity toward cancer cells with elevated lysosomal pH. We propose that the IDB drug targeting platform presents a paradigm shift in the approach of designing new anticancer agents with enhanced and optimized selectivity toward tumor cells. Traditional SAR approaches for enhancing selectivity focus mainly on designing drugs with greater potency against cancer cells, with little attention paid to the activity of the drug in normal cells. Most drug delivery-based approaches that attempt to increase selectivity rely on tumor-specific drug accumulation/delivery, which has proven to be quite challenging and difficult to attain. The IDB drug targeting approach described here allows the drug to distribute equally to both normal and transformed cells; however, the drug therapeutic index is widened by purposefully designing the drug to have unfavorable intracellular distribution properties in normal cells, thus reducing drug activity in these cells.

Our previous study had established that lysosomotropic molecules had measurable IDB selectivity, but it remained unclear whether the degree of lysosomotropism correlated with the degree of IDB selectivity. Our hypothesis leading into this study was that anticancer agents with optimal lysosomotropic properties would have optimal IDB selectivity. We initially utilized ion trapping principles to theoretically establish the relationships between weak base properties of drugs, specifically permeability (alpha) and pKa, and lysosomal sequestration (see Figure 1). These calculations were revealing and suggested that weakly basic molecules with low alpha and pKa near or above neutrality would have optimal lysosomotropic properties.

Our theoretical predictions illustrate that the alpha permeability parameter represents an important predictor of lysosomal sequestration; however, it is not a physical parameter that can be rationally and systematically changed to test the relationship between lysosomotropism and IDB selectivity. We have previously experimentally estimated alpha by measuring octanol/water partition coefficients as a function of pH and found that molecules either tended to have alpha parameters near zero or near one [7]. Although alpha can vary from zero to one, our theoretical predictions revealed that relatively small changes in the values of alpha near zero had a profound impact on the relationship between weak base pKa and the magnitude of lysosomal sequestration (Figure 1). It is currently unclear what structural characteristics might be responsible for such minor perturbations in the alpha parameter (i.e., 0.01 versus 0.001).

Unlike the alpha parameter, pKa represents a physical property that can be rationally modified to test the relationship between lysosomotropism and selectivity. To systematically investigate the influence of weak base pKa on IDB selectivity, we synthesized a small library of analogs of GDA generated by modifying GDA at the 17-position. These GDA analogs were particularly well-suited for such an application because numerous analogs formed by synthetic modifications at this position have been shown to retain their ability to bind with and inhibit Hsp90 [25]. In addition, GDA has a cytosolic target, and therefore, the activity of GDA analogs should be sensitive to changes in lysosomal sequestration. We synthesized weakly basic analogs of GDA with pKa values that varied from 5.8 to 12.4, and we observed that they all appeared to retain some capacity to bind rHsp90 as unmodified GDA (Figure 2). It is important to note that differences in intrinsic Hsp90 binding affinity among the various derivatives may indeed influence the magnitude of an experimentally observed IC_50_ value in a single cell line; however, these differences should not theoretically influence our selectivity comparisons presented in Figures 3 and 5. This is because we report the ratio of IC_50_ values for a single drug in cells with low or elevated lysosomal pH, thus factoring out any differences in intrinsic activity that may be present.

Use of shRNA to create variants of the MDA-MB-231 cell lines that differed in lysosomal pH represents a valuable tool to directly test IDB selectivity. In our previously published evaluations, we utilized comparative IC_50_ evaluations in two different cell lines with low or elevated lysosomal pH [12]. In addition, we utilized a pharmacological modulator of lysosomal pH (i.e., ammonium chloride) to achieve lysosomal pH elevation. Both of these approaches have limitations because the differences in cytotoxicity could be attributed to the differences in cell lines or to the addition of the pH modifier and not specifically to the differences in lysosomal pH.

The GDA analog with a measured pKa of 8.1 was found to have the maximum degree of IDB selectivity. GDA analogs with pKa values well above or below 8 showed minimal selectivity, which is consistent with the notion that lysosomotropic potential correlates with IDB selectivity. To assess the overall selectivity of the GDA analogs, we compared the ratio of IC_50_ values in normal fibroblasts versus in HL-60 leukemic cells, which have been shown to have elevated lysosomal pH [22]. Interestingly, the trend in the overall selectivity of the GDA analogs was similar in appearance and magnitude with the IDB selectivity results. This finding suggests that IDB selectivity can represent a major contributing factor towards overall selectivity.

To further evaluate the influence of lysosomotropism on IDB selectivity, we probed the correlation using currently available anticancer agents that had different structures, intracellular targets and mechanisms of action from those of the previously evaluated Hsp90 inhibitors (Figure 5). Consistent with our hypothesis, the non-lysosomotropic anticancer agents 5-fluorouracil and chlorambucil did not show appreciable IDB selectivity. In contrast, weakly basic anticancer agents with pKa values near 8 were found to possess IDB selectivity. Specifically, the weakly basic drugs mitoxantrone and daunorubicin, which have pKa values of 8.1 and 8.4, respectively, were both found to possess significant degrees of IDB selectivity. It is interesting to note that daunorubicin had a significantly higher IDB selectivity than did mitoxantrone. This could be attributed to the fact that the pKa of daunorubicin is slightly higher than the pKa of mitoxantrone and that relatively minor changes in pKa can translate into fairly significant increases in IDB selectivity. It is also plausible that there could be slight variations in the alpha permeability parameter for these two compounds that could result in dramatic alterations in lysosomotropism as was theoretically determined (Figure 1). In addition to these drugs, numerous other clinically approved anticancer agents can be classified as weakly basic molecules. To our knowledge, there is currently no clinical evidence to support the notion that their weakly basic properties contributes to their observed selectivity.

It is important to emphasize that the IDB drug targeting platform described here will not be universally applicable to all cancer types. An obvious prerequisite to the successful application is that the cancer cells have a lysosomal acidification defect. We and others have shown that many different cancer cell types have defective lysosomal pH [19]–[22]; however, as we have illustrated in this manuscript, some cancer cells, such as the MBA-MB-231 cell line, have lysosomal pH values that are similar to those in normal cells. In addition, some work has suggested that the emergence of drug resistance of cancer cells is associated with the re-acidification of lysosomes [35]. More work will be needed to identify the prevalence and mechanism of defective acidification in primary human tumors.

Another important consideration regarding the potential applicability of the IDB targeting platform is the pH of the tumor microenvironment. In most solid tumors, the extracellular pH can be quite acidic [36]. This might also be the case for leukemic stem cells localized in hypoxic niches in the bone marrow. Anticancer agents with pKa values near 8 will be more extensively ionized in the extracellular fluid surrounding such tumor cells than in the extracellular fluid surrounding normal cells. Such differences in extracellular ionization could reduce the kinetics of accumulation of weakly basic drugs in tumor cells compared to normal cells and partially or fully offset any benefits derived from IDB selectivity. However, hematological cancers as well as circulating metastatic malignancies would not experience such extracellular acidification. Accordingly, the results of this work may be most applicable to such freely circulating cancer cells. Future examinations with additional cancer cell types (i.e., metastatic malignancies) with variation in the extracellular pH of the cell culture media might further define the limitations and applicability of this approach to enhancing selectivity.

In conclusion, we propose that the findings reported in this study have significant implications toward the rational synthesis of new drugs that have optimized selectivity toward transformed cells or the modification of existing drugs. Specifically, our results suggest that medicinal chemistry-based efforts that promote lysosomotropism of anticancer agents might increase their therapeutic index. However, there are potential downsides associated with the development of highly lysosomotropic drugs that need to be considered and could not be fully explored using our cell culture-based evaluations. Specifically, several cationic amphiphillic drugs that extensively accumulate within lysosomes have been shown to cause a lysosomal phospholipidosis *in vivo*, potentially through their inhibition of lysosomal phospholipases [37]. The long-term toxicological implications of drug-induced phospholipidosis, if any, remain to be elucidated. In addition, it is possible that imparting lysosomotropic properties might negatively impact other pharmacokinetic attributes and/or the propensity for off-target interactions unrelated to lysosomal sequestration. Medicinal chemists involved in early drug discovery strive to optimize the structural and physicochemical properties of drugs to promote high affinity interactions with intended targets and to make the candidate possess “drug-like” properties to minimize attrition during the various stages of development [38]–[40]. Such failures are often attributed to poor ADMET (absorption, metabolism, metabolism, elimination and transport) properties and/or unacceptable toxicity profiles [41]. Accordingly, one must recognize that optimization of basicity may be beneficial for selectivity, as proposed here, but such alterations may adversely impact other important attributes that could potentially cause failure. Ultimately, a holistic approach is required to develop a successful drug.

## Materials and Methods

### Cell lines and cell culture reagents

HL-60 cells were kindly provided by Dr. Yuesheng Zhang and were propagated as described [42]. Normal human fibroblasts (CRL-2076), MDA-MB-231 (HTB-26) human breast adenocarcinoma cells and HEK 293T cells were obtained from the American Type Culture Collection (Rockland, MD) and were propagated according to the supplier's guidelines. All cells were maintained at 37°C in a humidified incubator supplied with 5% CO_2_, and the cells were passaged for up to 2 months.

### Synthesis of geldanamycin analogs varying in pKa

Geldanamycin was obtained from LC Labs (Woburn, MA). All other chemicals were obtained from Sigma Aldrich (St. Louis, MO) or Alfa Aesar (Ward Hill, MA) and were used as received. DMF was freshly distilled and dried over a 3Å molecular sieve before use. The following synthetic methods were followed:

#### Synthetic method A

To a solution of GDA (1 eq. in DMF) was added the corresponding amine (2 eq.) dissolved in DMF, and the mixture was stirred at room temperature for 24 h in the dark. Afterwards, the reaction mixture was diluted with water and washed three times with chloroform. The organic layers were combined and dried over anhydrous Na_2_SO_4_. The solvent was then removed under reduced pressure, and the crude product was purified by flash chromatography with silica using methanol/chloroform as eluent.

#### Synthetic method B

Sodium (10 eq. of 60% w/w in mineral oil) was added to the corresponding amine (5 eq.) dissolved in DMF. The mixture was stirred at room temperature under argon for 20 min, followed by the addition of GDA (1 eq.) in DMF. After 24 h, DMF was removed under reduced pressure, and the crude product was purified by recrystallization from methanol/chloroform.

The GDA analogs were characterized using ^1^H-NMR, and the ^1^H-NMR data are shown in Text S1. The specific amines used for the syntheses are also included in the supplementary information.

### Determination of pKa values of geldanamycin analogs

The ionization constants of GDA analogs were determined by recording the pH-dependent chemical shift of methylene protons alpha to the weakly basic amine, as described previously [43], [44]. All NMR solvents and reagents were obtained from Cambridge Isotope Labs (Andover, MA), unless noted otherwise. Compounds were dissolved at a 2–3 mg/mL concentration in D_2_O. pD measurements in D_2_O were performed using a MiniLab model IQ125 pH electrode (Fisher Scientific, Pittsburg, PA). pD was adjusted using NaOD (40% in D_2_O) or DCl (20% in D_2_O). ^1^H NMR spectra were recorded at 37°C using a Bruker 400 MHz NMR and analyzed using the Bruker Topspin software. 4-Dimethyl-4-silapentane-1-sulfonic acid (DSS) was used as the reference peak. pD was converted to pH using the following expression: pH = 0.936pD*+0.412 [45]. The change in chemical shift of protons alpha to the amine as a function of pH yielded sigmoidal plots that were fit to a 4-parameter logistic equation using SigmaPlot (Systat Software, San Jose, CA) from which pKa was determined. The calculated pKa for compound 7 was determined using the MarvinSketch version 5.3.8. pKa plugin (www.chemaxon.com).

### Assessment of Hsp90 binding affinity of geldanamycin analogs

The binding affinity of the weakly basic GDA analogues to rHsp90 was measured using a previously described competitive displacement fluorescence polarization assay [46], using GDA conjugated to FITC (BioMol, Enzo Life Sciences, Plymouth Meeting, PA) as the fluorescent tracer molecule. Non-binding surface 96-well plates (Corning) used for the binding experiments were obtained from Fisher Scientific. A binding buffer composed of 20 mM Hepes, pH 7.3, 50 mM KCl, 5 mM MgCl_2_, 20 mM Na_2_MoO_4_, and 0.01% NP40 was used. Bovine gamma globulin (0.1 mg/mL) and 1 mM DTT were freshly added to the buffer before use. GDA-FITC was reconstituted in DMSO at a stock concentration of 10 µM and was used at a final concentration of 2 nM. The rHsp90 (Enzo Life Sciences) was diluted to a final concentration of 40 nM. Eight different concentrations of GDA analogs ranging from 0 to 2000 nM were added to a mixture of rHsp90 and GDA-FITC in individual wells of a 96-well plate, and the plates were placed on a shaker in a 4°C refrigerator for 4 h. Fluorescence polarization measurements were performed using a Wallac Envision Multilabel plate reader (Perkin Elmer), as previously described [47]. Plots of fluorescence polarization versus GDA analog concentration yielded sigmoidal curves that were fit to 3 or 4 parameter logistic equations from which the IC_50_ values were obtained. The IC_50_ values obtained from the plots were converted to K_i_ values as previously described [28].

### Generation of MD-MBA-231 cells with elevated lysosomal pH

MDA-MB-231 cells with elevated lysosomal pH were generated by lentiviral vector-mediated knockdown of the V-ATPase V1E1 subunit (ATP6V1E1). The lentiviral transfer vector pPGK-Neo-ATP6V1E1 encoding the target sequence 5′-CAGATGTCTCCAATTTGATGAAT-3′ against the V-ATPase V1E1 subunit was obtained from Mission (Sigma, St. Louis, MO). The control cells were treated with a pPGK-Neo transfer vector containing the sequence 5′-CAACAAGATGAAGAGCACCAA-3′, which does not target any known mammalian gene. Lentiviral particles were produced by transfecting HEK 293T cells with the transfer vector together with a proprietary lentiviral packaging mix from Sigma, following the manufacturer's instructions. For the transfection, 5×10^5^ HEK 293T cells were seeded in 10 cm dishes and allowed to adhere overnight. After the cells adhered, 2.6 µg of vector DNA was combined with 26 µL of the lentiviral packaging mix and transfected into cells using Fugene-6 transfection reagent (Roche Diagnostics, Indianapolis, IN), according to the manufacturer's instructions. The transfection complexes were incubated with the HEK 293T cells overnight and then the media was replaced. The virus-containing cell culture supernatants were harvested twice: once at 24 h and again at 48 h. Prior to use, the viral media was filtered through a 0.45-µm filter and added onto 70% confluent 75 cc flasks of MDA-MB-231 cells, in the presence of 8 µg/mL hexadimethrine bromide. The viruses were allowed to infect the cells for 16–18 h, after which the media containing viruses was replaced with fresh media for a further 24 h prior to analysis of knockdown of V1E1 expression. The knockdown efficiency was analyzed by Western blotting, performed as described previously [22]. A goat polyclonal antibody to the V1E1 subunit (sc-21218; Santa Cruz Biotechnology, Santa Cruz, CA) was used at a 1∶200 dilution to detect VIE1, and a mouse monoclonal antibody was used at a 1∶10000 dilution to detect actin (loading control). Anti-goat HRP-conjugated secondary antibody (Santa Cruz Biotechnology) was used at a 1∶5000 dilution, and anti-mouse HRP-conjugated secondary antibody was used at a 1∶1000 dilution.

### Determination of lysosomal pH

Lysosomal pH was determined following a previously a published protocol [24], with modifications. Briefly, 1×10^5^ cells/well were plated in 8-chamber tissue culture treated microscope slides (BD Falcon, Bedford, MA) and allowed to adhere overnight. Cells were incubated with 1 mg/mL Oregon Green 488 dextran 10,000 MW (Invitrogen) for 2 h, followed by 6 h incubation in dextran-free medium to allow the dextran to specifically localize in terminal lysosomes. At the end of the chase period, the cells were rinsed with and maintained in lysosomal pH buffer composed of150 mM NaCl, 20 mM Mes, 5 mM KCl, and 1 mM MgSO_4_, pH 7.4. The emission of Oregon Green 488 at both 495 and 450 nm excitation wavelengths at 525/10 nm emission in the cells was then measured using a microscope-adapted Ratiomaster spectrofluorimeter (PTI, Trenton, NJ) equipped with a photomultiplier tube detector. The ratio of emission at 495 nm excitation to the emission at 450 nm excitation was calculated and converted into lysosomal pH using a lysosomal pH calibration curve. To generate the lysosomal pH calibration curve, lysosomal pH buffers of pH 4, 5, 5.5, and 6 containing 10 µM nigericin and 20 µM monensin were added to respective wells of the 8-chamber slide for 30 min to equilibrate intracellular pH with extracellular pH, and the fluorescence emission was measured as described above. The emission intensity ratios were calculated as described, and a standard curve was obtained by plotting pH against the respective 495/450 nm emission ratios. The calibration curve was linear between pH 4–6.

### Antiproliferative evaluations for anticancer agents

Cell sensitivity to compounds was evaluated using the WST-1 (4-[3[(4-iodophenyl)-2-(4-nitrophenyl)-2H-5-tetrazolio]-1,3-benzene disulfonate) assay (BioAssay Systems, Hayward, CA), which was performed according to the manufacturer's instructions. Briefly, 4000 cells were seeded per well of a 96-well plate and allowed to adhere overnight. Cells were incubated in increasing concentrations (9 different concentrations ranging from 0.00001 µM to 1000 µM) of drug for a period of 72 h. At the end of the drug treatment period, 10 µL of WST-1 reagent was added per well, allowed to incubate at 37°C for 2 hours, and absorbance at 450 and 620 nm (reference) was measured using a Multiskan model MCC/340 microplate reader (ThermoElectron Corp, Hudson, NH). Cell viability as a percent of control (untreated) cells was plotted against drug concentration to generate cytotoxicity curves, which were fit to 3 or 4 parameter logistic Hill plots using SigmaPlot. IC_50_ values were determined from the curve fit.

### Statistical Analysis

Data presentation and statistical analysis was carried out using Sigma Plot v10.0 and v12.3 (SPSS, Chicago, IL). Statistical analyses of significance (*p* values) were calculated using Students' *t*-tests comparing the selectivity values of the GDA analogs with those of GDA. Results were considered statistically significant when *p*<0.05.

## Supporting Information

Figure S1 **pH-dependent chemical shifts of protons adjacent to the amine group of geldanamycin analogs at 37°C.** The chemical shifts were plotted against pH and fit to a 3-parameter sigmoidal curve fit (solid line). pKa was determined from the curve fit.
(TIF)

Figure S2 **Competitive inhibition of geldanamycin-FITC binding to rHsp90 by geldanamycin and analogs.** Competitive inhibition was measured using a fluorescence polarization assay. Polarization values of drug-treated wells were converted to percent of control wells (no drug) and plotted against drug concentration. The curves were fit to 3- or 4-parameter logistic Hill plots from which the IC_50_ was determined. The data points are the mean of duplicate experiments.
(TIF)

Figure S3 **Cytotoxicity of geldanamycin and analogs in normal fibroblasts and in HL60 human leukemic cells.** Cells were exposed to the indicated varying concentrations of GDA and its analogs for 72 h. The filled circles represent data acquired from normal fibroblasts and the open circles represent data from experiments with HL60 cells. The IC_50_ was determined from the curve fit using 3- or 4-parameter logistic Hill plots. Data represent mean±SD (n = 3).
(TIF)

Figure S4 **Cytotoxicity of geldanamycin and its analogs in MDA-MB 231 cells with low and elevated lysosomal pH.** The cells were exposed to the indicated concentrations of GDA and its analogs for 72 h. The filled circles represent scrambled shRNA-treated cells with low lysosomal pH and the open circles represent V1E1 shRNA-treated cells with elevated lysosomal pH. The IC_50_ was determined by fitting the curves to 3- or 4-parameter logistic Hill plots. Data represent mean±SD (n = 3).
(TIF)

Figure S5 **Cytotoxicity of anticancer agents in MDA-MB 231 cells with low and elevated lysosomal pH.** The cells were exposed to indicated concentrations of mitoxantrone, daunorubicin, 5-fluorouracil and chlorambucil for 72 h. The filled circles represent data from cells treated with scrambled shRNA with low lysosomal pH and the open circles represent data from V1E1 shRNA-treated cells with elevated lysosomal pH. The IC_50_ was determined by fitting the curves to 3- or 4-parameter logistic Hill plots. Data represent mean±SD (n = 3).
(TIF)

Text S1 Method of synthesis and ^1^H-NMR characterization of geldanamycin analogs. Geldanamycin analogs were synthesized using Method A or B, as described in Materials and Methods, and characterized by ^1^H-NMR.
(DOCX)

## References

[1] Kroemer G, Pouyssegur J (2008) Tumor cell metabolism: cancer's Achilles' heel. Cancer Cell 13: 472–482. 18538731 10.1016/j.ccr.2008.05.005

[2] Dutcher JP, Novik Y, O'Boyle K, Marcoullis G, Secco C, et al (2000) 20th-century advances in drug therapy in oncology–Part I. J Clin Pharmacol. 40: 1007–1024. 10.1177/00912700022009620 10975071

[3] Dutcher JP, Novik Y, O'Boyle K, Marcoullis G, Secco C, et al (2000) 20th-century advances in drug therapy in oncology–Part. II. J Clin Pharmacol 40: 1079–1092. 11028247

[4] Imai K, Takaoka A (2006) Comparing antibody and small-molecule therapies for cancer. Nat Rev Cancer 6: 714–727. 16929325 10.1038/nrc1913

[5] Houshmand P, Zlotnik A (2003) Targeting tumor cells. Curr Opin Cell Biol 15: 640–644. 14519400 10.1016/s0955-0674(03)00106-6

[6] Petrak K (2005) Essential properties of drug-targeting delivery systems. Drug Discov Today 10: 1667–1673. 16376827 10.1016/S1359-6446(05)03698-6

[7] Duvvuri M, Gong Y, Chatterji D, Krise JP (2004) Weak base permeability characteristics influence the intracellular sequestration site in the multidrug-resistant human leukemic cell line HL-60. J Biol Chem 279: 32367–32372. 15181006 10.1074/jbc.M400735200

[8] Duvvuri M, Konkar S, Funk RS, Krise JM, Krise JP (2005) A chemical strategy to manipulate the intracellular localization of drugs in resistant cancer cells. Biochemistry 44: 15743–15749. 16313177 10.1021/bi051759w

[9] Trapp S, Rosania G, Horobin R, Kornhuber J (2008) Quantitative modeling of selective lysosomal targeting for drug design. Eur Biophys J 37: 1317–1328. 18504571 10.1007/s00249-008-0338-4 PMC2711917

[10] Egorin MJ, Clawson RE, Cohen JL, Ross LA, Bachur NR (1980) Cytofluorescence localization of anthracycline antibiotics. Cancer Res 40: 4669–4676. 6934029

[11] Kaufmann AM, Krise JP (2008) Niemann-Pick C1 functions in regulating lysosomal amine content. J Biol Chem 283: 24584–24593. 18591242 10.1074/jbc.M803715200 PMC2528997

[12] Duvvuri M, Konkar S, Hong KH, Blagg BS, Krise JP (2006) A new approach for enhancing differential selectivity of drugs to cancer cells. ACS Chem Biol 1: 309–315. 17163760 10.1021/cb6001202

[13] Kang BH, Altieri DC (2009) Compartmentalized cancer drug discovery targeting mitochondrial Hsp90 chaperones. Oncogene 28: 3681–3688. 19648961 10.1038/onc.2009.227 PMC2766018

[14] Lansiaux A, Tanious F, Mishal Z, Dassonneville L, Kumar A, et al (2002) Distribution of furamidine analogues in tumor cells: targeting of the nucleus or mitochondria depending on the amidine substitution. Cancer Res 62: 7219–7229. 12499262

[15] Fernandez-Carneado J, Van Gool M, Martos V, Castel S, Prados P, et al (2005) Highly efficient, nonpeptidic oligoguanidinium vectors that selectively internalize into mitochondria. J Am Chem Soc 127: 869–874. 15656624 10.1021/ja044006q

[16] Kang BH, Tavecchio M, Goel HL, Hsieh CC, Garlick DS, et al (2011) Targeted inhibition of mitochondrial Hsp90 suppresses localised and metastatic prostate cancer growth in a genetic mouse model of disease. Brit J Cancer 104: 629–634. 21285984 10.1038/bjc.2011.9 PMC3049604

[17] de Duve C, de Barsy T, Trouet A, Tulkens P, van Hoof F (1974) Lysosomotropic agents. Biochem Pharmacol 23: 2495–2531. 4606365 10.1016/0006-2952(74)90174-9

[18] Bulychev A, Trouet A, Tulkens P (1978) Uptake and intracellular distribution of neutral red in cultured fibroblasts. Exp Cell Res 115: 343–355. 689090 10.1016/0014-4827(78)90288-4

[19] Jiang LW, Maher VM, McCormick JJ, Schindler M (1990) Alkalinization of the lysosomes is correlated with ras transformation of murine and human fibroblasts. J Biol Chem 265: 4775–4777. 1690732

[20] Altan N, Chen Y, Schindler M, Simon SM (1998) Defective acidification in human breast tumor cells and implications for chemotherapy. J Exp Med 187: 1583–1598. 9584137 10.1084/jem.187.10.1583 PMC2212293

[21] Kokkonen N, Rivinoja A, Kauppila A, Suokas M, Kellokumpu I, et al (2004) Defective acidification of intracellular organelles results in aberrant secretion of cathepsin D in cancer cells. J Biol Chem 279: 39982–39988. 15258139 10.1074/jbc.M406698200

[22] Gong Y, Duvvuri M, Krise JP (2003) Separate roles for the Golgi apparatus and lysosomes in the sequestration of drugs in the multi-drug resistant human leukemic cell line HL-60. J Biol Chem 278: 50234–50239. 14522995 10.1074/jbc.M306606200

[23] Ndolo RA, Jacobs DT, Forrest ML, Krise JP (2010) Intracellular distribution-based anticancer drug targeting: Exploiting a lysosomal acidification defect associated with cancer cells. Mol Cell Pharmacol 2: 131–136. 21274418 10.4255/mcpharmacol.10.18 PMC3026327

[24] Bocchetta M, Eliasz S, De Marco MA, Rudzinski J, Zhang L, et al (2008) The SV40 large T antigen-p53 complexes bind and activate the insulin-like growth factor-I promoter stimulating cell growth. Cancer Res 68: 1022–1029. 18281476 10.1158/0008-5472.CAN-07-5203

[25] Tian Z-Q, Liu Y, Zhang D, Wang Z, Dong SD, et al (2004) Synthesis and biological activities of novel 17-aminogeldanamycin derivatives. Bioorg Med Chem 12: 5317–5329. 15388159 10.1016/j.bmc.2004.07.053

[26] Duvvuri M, Krise JP (2005) A novel assay reveals that weakly basic model compounds concentrate in lysosomes to an extent greater than pH-partitioning theory would predict. Mol Pharm 2: 440–448. 16323951 10.1021/mp050043s

[27] Nilsson C, Kågedal K, Johansson U, Öllinger K (2004) Analysis of cytosolic and lysosomal pH in apoptotic cells by flow cytometry. Methods Cell Sci 25: 185–194. 10.1007/s11022-004-8228-3 15801164

[28] Nikolovska-Coleska Z, Wang R, Fang X, Pan H, Tomita Y, et al (2004) Development and optimization of a binding assay for the XIAP BIR3 domain using fluorescence polarization. Anal Biochem 332: 261–273. 15325294 10.1016/j.ab.2004.05.055

[29] Howes R, Barril X, Dymock BW, Grant K, Northfield CJ, et al (2006) A fluorescence polarization assay for inhibitors of Hsp90. Anal Biochem 350: 202–213. 16460658 10.1016/j.ab.2005.12.023

[30] Young JC, Moarefi I, Hartl FU (2001) Hsp90: a specialized but essential protein-folding tool. J Cell Biol 154: 267–274. 11470816 10.1083/jcb.200104079 PMC2150759

[31] Lu X, Qin W, Li J, Tan N, Pan D, et al (2005) The growth and metastasis of human hepatocellular carcinoma xenografts are inhibited by small interfering RNA targeting to the subunit ATP6L of proton pump. Cancer Res 65: 6843–6849. 16061667 10.1158/0008-5472.CAN-04-3822

[32] Mahoney BP, Raghunand N, Baggett B, Gillies RJ (2003) Tumor acidity, ion trapping and chemotherapeutics: I. Acid pH affects the distribution of chemotherapeutic agents in vitro. Biochem Pharmacol 66: 1207–1218. 14505800 10.1016/s0006-2952(03)00467-2

[33] Milano G, Thyss A, Santini J, Frenay M, Francois E, et al (1989) Salivary passage of 5-fluorouracil during continuous infusion. Cancer Chemother Pharmacol 24: 197–199. 2736710 10.1007/BF00300243

[34] Adair CG, McElnay JC (1986) Studies on the mechanism of gastrointestinal absorption of melphalan and chlorambucil. Cancer Chemother Pharmacol 17: 95–98. 3698183 10.1007/BF00299875

[35] Schindler M, Grabski S, Hoff E, Simon SM (1996) Defective pH regulation of acidic compartments in human breast cancer cells (MCF-7) is normalized in adriamycin-resistant cells (MCF-7adr). Biochemistry 35: 2811–2817. 8608115 10.1021/bi952234e

[36] Gillies RJ, Liu Z, Bhujwalla Z (1994) 31P-MRS measurements of extracellular pH of tumors using 3-aminopropylphosphonate. Am J Physiol - Cell Physiol 267: C195–C203. 10.1152/ajpcell.1994.267.1.C195 8048479

[37] Reasor MJ, Kacew S (2001) Drug-induced phospholipidosis: are there functional consequences? Exp Biol M 226: 825–830. 10.1177/153537020122600903 11568304

[38] Bickerton GR, Paolini GV, Besnard J, Muresan S, Hopkins AL (2012) Quantifying the chemical beauty of drugs. Nat Chem 4: 90–98. 22270643 10.1038/nchem.1243 PMC3524573

[39] Hann MM, Keseru GM (2012) Finding the sweet spot: the role of nature and nurture in medicinal chemistry. Nat Rev Drug Discov 11: 355–365. 22543468 10.1038/nrd3701

[40] Lipinski CA, Lombardo F, Dominy BW, Feeney PJ (2001) Experimental and computational approaches to estimate solubility and permeability in drug discovery and development settings. Adv Drug Deliver Rev 46: 3–26. 10.1016/s0169-409x(00)00129-0 11259830

[41] Meanwell NA (2011) Improving drug candidates by design: a focus on physicochemical properties as a means of improving compound disposition and safety. Chem Res Toxicol 24: 1420–1456. 21790149 10.1021/tx200211v

[42] Liao Z, Lin J, Luo D (2008) Expressions and significance of uPA and V-ATPase mRNA in hepatocellular carcinoma. Chin-Germ J Clin Oncol 7: 631–634.

[43] Szakács Z, Hägele G (2004) Accurate determination of low pK values by 1H NMR titration. Talanta 62: 819–825. 18969368 10.1016/j.talanta.2003.10.007

[44] Grycová L, Dommisse R, Pieters L, Marek R (2009) NMR determination of pKa values of indoloquinoline alkaloids. Magn Res Chem 47: 977–981. 10.1002/mrc.2494 19653253

[45] Krezel A, Bal W (2004) A formula for correlating pKa values determined in D2O and H2O. J Inorg Biochem 98: 161–166. 14659645 10.1016/j.jinorgbio.2003.10.001

[46] Llauger-Bufi L, Felts SJ, Huezo H, Rosen N, Chiosis G (2003) Synthesis of novel fluorescent probes for the molecular chaperone Hsp90. Bioorg Med Chem Lett 13: 3975–3978. 14592488 10.1016/j.bmcl.2003.08.065

[47] Borrok MJ, Kiessling LL (2007) Non-carbohydrate inhibitors of the lectin DC-SIGN. J Am Chem Soc 129: 12780–12785. 17902657 10.1021/ja072944v PMC2546503

