# Supplementary figures and images for: Lysosomotropic Properties of Weakly Basic Anticancer Agents Promote Cancer Cell Selectivity In Vitro
Source: PLoS One. 2012 Nov 7;7(11):e49366. doi: 10.1371/journal.pone.0049366 (PMC3492287; doi:10.1371/journal.pone.0049366)

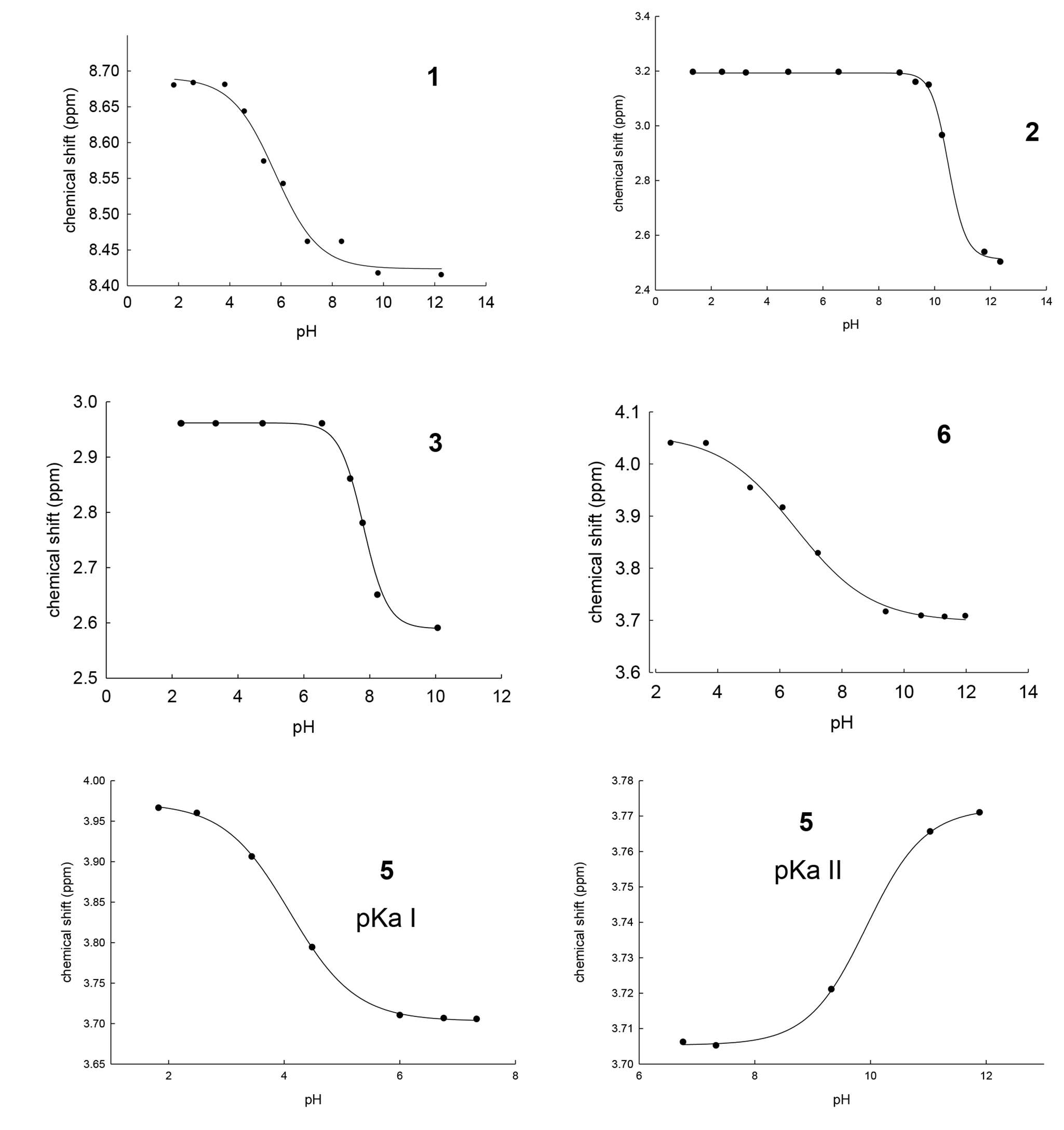

Supplement: Figure S1 — pH-dependent chemical shifts of protons adjacent to the amine group of geldanamycin analogs at 37°C. The chemical shifts were plotted against pH and fit to a 3-parameter sigmoidal curve fit (solid line). pKa was determined from the curve fit. (TIF) [file pone.0049366.s001.tif]

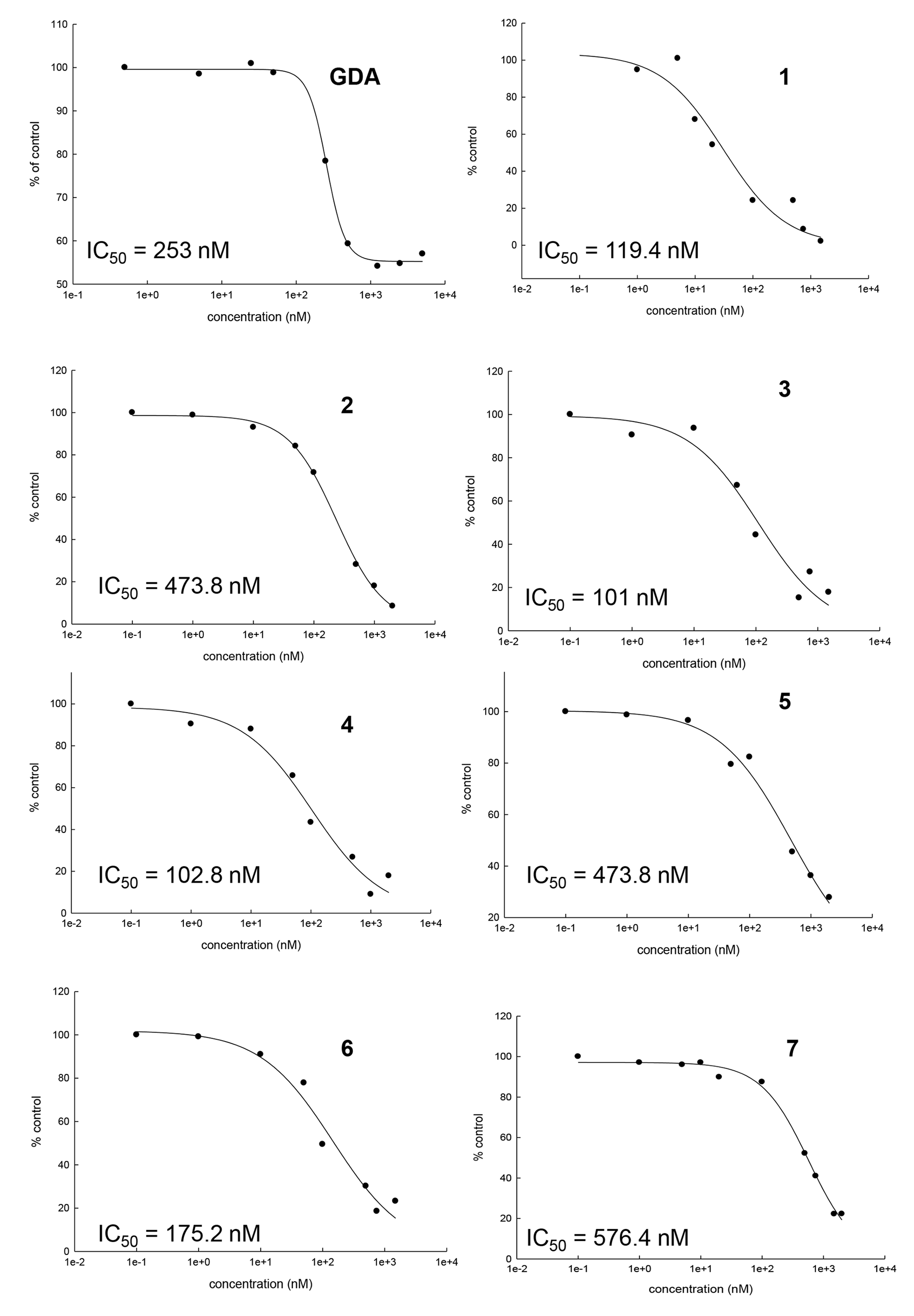

Supplement: Figure S2 — Competitive inhibition of geldanamycin-FITC binding to rHsp90 by geldanamycin and analogs. Competitive inhibition was measured using a fluorescence polarization assay. Polarization values of drug-treated wells were converted to percent of control wells (no drug) and plotted against drug concentration. The curves were fit to 3- or 4-parameter logistic Hill plots from which the IC50 was determined. The data points are the mean of duplicate experiments. (TIF) [file pone.0049366.s002.tif]

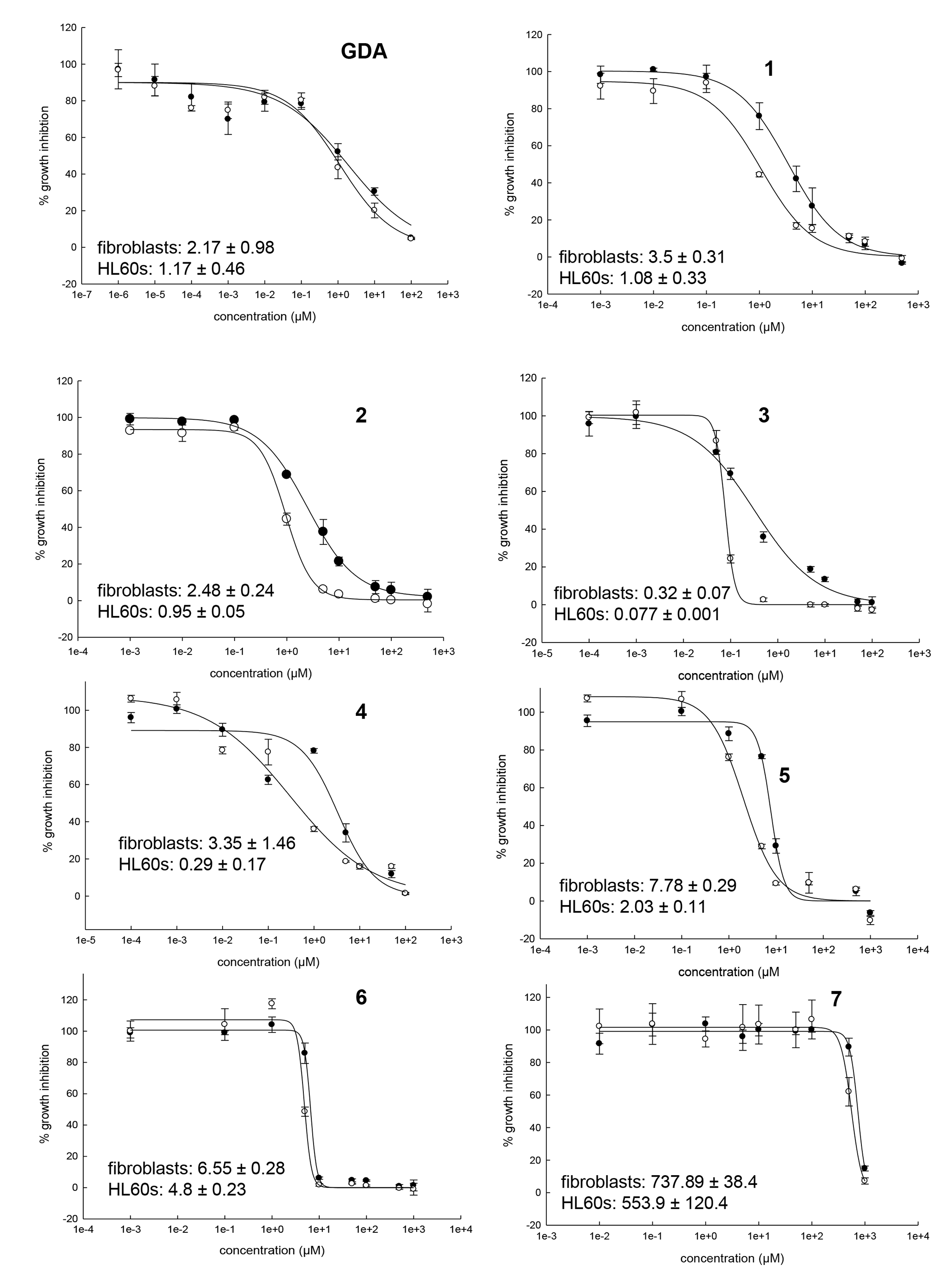

Supplement: Figure S3 — Cytotoxicity of geldanamycin and analogs in normal fibroblasts and in HL60 human leukemic cells. Cells were exposed to the indicated varying concentrations of GDA and its analogs for 72 h. The filled circles represent data acquired from normal fibroblasts and the open circles represent data from experiments with HL60 cells. The IC50 was determined from the curve fit using 3- or 4-parameter logistic Hill plots. Data represent mean±SD (n = 3). (TIF) [file pone.0049366.s003.tif]

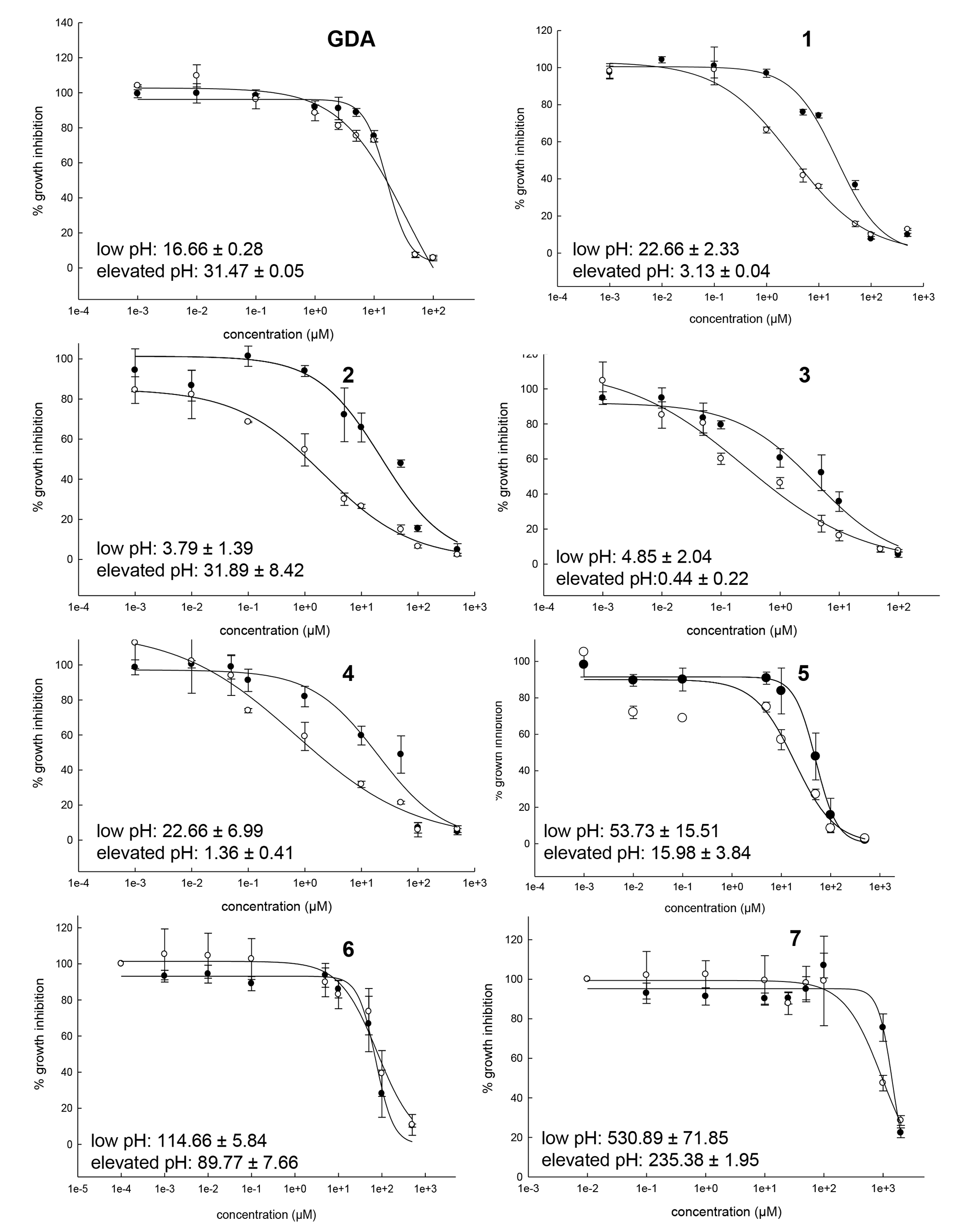

Supplement: Figure S4 — Cytotoxicity of geldanamycin and its analogs in MDA-MB 231 cells with low and elevated lysosomal pH. The cells were exposed to the indicated concentrations of GDA and its analogs for 72 h. The filled circles represent scrambled shRNA-treated cells with low lysosomal pH and the open circles represent V1E1 shRNA-treated cells with elevated lysosomal pH. The IC50 was determined by fitting the curves to 3- or 4-parameter logistic Hill plots. Data represent mean±SD (n = 3). (TIF) [file pone.0049366.s004.tif]

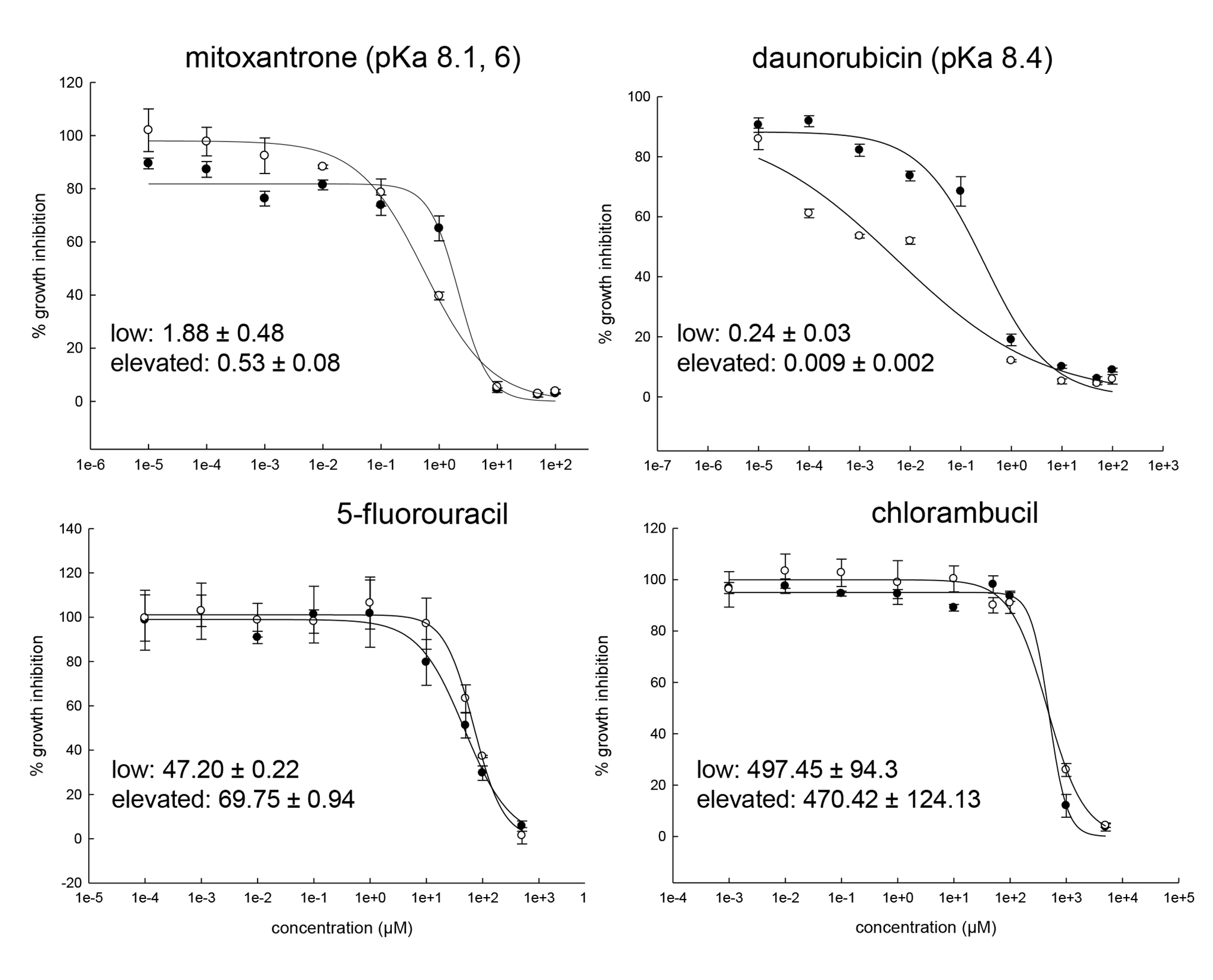

Supplement: Figure S5 — Cytotoxicity of anticancer agents in MDA-MB 231 cells with low and elevated lysosomal pH. The cells were exposed to indicated concentrations of mitoxantrone, daunorubicin, 5-fluorouracil and chlorambucil for 72 h. The filled circles represent data from cells treated with scrambled shRNA with low lysosomal pH and the open circles represent data from V1E1 shRNA-treated cells with elevated lysosomal pH. The IC50 was determined by fitting the curves to 3- or 4-parameter logistic Hill plots. Data represent mean±SD (n = 3). (TIF) [file pone.0049366.s005.tif]
