# Supplementary material for: Lysosomotropic Properties of Weakly Basic Anticancer Agents Promote Cancer Cell Selectivity In Vitro
Source: PLoS One. 2012 Nov 7;7(11):e49366. doi: 10.1371/journal.pone.0049366 (PMC3492287; doi:10.1371/journal.pone.0049366)
Supplement: Text S1 — Method of synthesis and 1H-NMR characterization of geldanamycin analogs. Geldanamycin analogs were synthesized using Method A or B, as described in Materials and Methods, and characterized by 1H-NMR. (DOCX) [file pone.0049366.s006.docx]

**^1^H-NMR Characterization of geldanamycin analogs**

**Compound 1**: 17-[2-(Pyridine-3-yl)ethyl]amino-17-demethoxygeldanamycin was synthesized following Method A and was obtained as a purple solid from geldanamycin and 3-pyridineethanamine, with a yield of 40%. ^1^H NMR (400 MHz, CDCl_3_): δ= 0.95 (d, 3H, J = 6.52 Hz), 1.01 (d, 3H, J = 6.92 Hz), 1.77 (brs, 1H), 1.78–1.80 (m, 3H), 1.81 (s, 3H), 2.04 (s, 3H), 2.39 (dd, 1H, J = 10.8, 13.6 Hz), 2.72 (d, 1H, J = 14.5 Hz), 2.75–2.78 (m, 1H), 2.99 (t, 1H, J = 6.92 Hz), 3.28 (s, 3H), 3.37 (s, 3H), 3.45 (m, 1H), 3.55–3.60 (m, 1H), 3.76 (m, 1H), 3.86 (m, 1H), 4.15 (br s, 1H), 4.31 (d, 1H, J = 9.76 Hz), 4.89 (br s, 2H), 5.20 (s, 1H), 5.87 (t, 1H, J = 10.6 Hz), 5.88 (d, 1H, J = 9.6 Hz), 6.29 (t, 1H, J = 5.52 Hz), 6.60 (t, 1H, J = 11.7), 6.97 (d, 1H, J = 11.6), 6.93 (d, 1H, J = 11.6Hz), 7.31 (m, 1H), 7.55 (m, 1H), 8.54 (m, 2H), 9.15 (s, 1H).

**Compound 2**: 17-[2-(Morpholin-4-yl)ethyl]amino-17-demethoxygeldanamycin was synthesized following Method A and was obtained as a purple solid from geldanamycin and 2-(4-morpholinyl)ethylamine, with a yield of 52%. ^1^H NMR (400 MHz, MeOD): δ=0.95 (d, 3H, J = 6.0 Hz), 1.00 (d, 3H, J = 7.96 Hz), 1.57–1.63 (m, 4H), 1.75 (s, 3H), 2.02 (s, 3H), 2.36(m, 1H), 2.53 (m, 4H), 2.66 (m, 2H), 2.67 – 2.78 (m, 2H), 2.88 (s, 3H), 3.01 (s, 3H), 3.44–3.52 (m, 2H), 3.61 (m, 1H), 3.68 (m, 1H), 3.72 (m, 4H), 4.12 (br s, 1H), 4.55 (d, 1H, J = 8.36 Hz), 4.99 (br s, 2H), 5.19 (s, 1H), 5.62 (d, 1H, J = 9.52 Hz), 5.89 (t, 1H, J = 8.92 Hz), 6.67 (br s, 1H), 7.01 (s, 1H), 7.17 (d, 1H, J = 11.2 Hz), 8.00 (s, 1H).

**Compound 3**: 17-(2-Dimethylaminoethylamino)-17-demethoxygeldanamycin was synthesized following Method A and was obtained as a purple solid from geldanamycin and 2-dimethylaminoethyl amine, with a yield of 40%. ^1^H NMR (400 MHz, CDCl_3_): *δ*= 0.99 (d, 3H, J = 6.5 Hz), 1.02 (d, 3H, J = 6.9 Hz), 1.67-1.81 (m, 3H), 1.82 (d, 3H, J = 1.1 Hz), 2.05 (d, 3H, J = 6.6 Hz), 2.29 (s, 6H), 2.44 (d, 1H, J = 11.2 Hz), 2.59 (t, 2H, J = 6.0 Hz), 2.71 (d, 1H, J = 13.9 Hz), 2.79-2.74 (m, 1H, J = 7.2 Hz), 3.29 (s, 3H), 3.39 (s, 3H), 3.52-3.47 (m, 2H), 3.62-3.58 (m, 1H), 3.73-3.67 (m, 1H), 4.33 (d, 1H, J = 10.0 Hz), 4.53 (br s, 1H), 4.80 (br s, 2H), 5.21 (s, 1H), 5.88 (t, 1H, J = 10.6 Hz), 5.94 (d, 1H, J = 9.5 Hz), 6.61 (t, 1H, J = 11.7 Hz), 6.98 (d, 1H, J = 12.9 Hz), 7.09 (br s, 1H), 7.27 (s, 1H), 9.21 (s, 1H).

**Compound 4**: 17-[2-(Pyrrolidin-1-yl)ethyl]amino-17-demethoxygeldanamycin was synthesized following Method A and was obtained as a purple solid from geldanamycin and 1-pyrrolidineethanamine, with a yield of 67%. ^1^H NMR (400 MHz, CDCl_3_): *δ*= 0.98 (d, 3H, J = 6.8 Hz), 1.01 (d, 3H, J = 6.9 Hz), 1.74 (brs, 1H), 1.76-1.82 (m, 9H), 2.05 (d, 3H, J = 6.6 Hz), 2.41 (d, 1H, J = 11.1 Hz), 2.57 (brs, 4H), 2.69 (d, 1H, J = 13.9 Hz), 2.82-2.72 (m, 3H), 3.45 (s, 3H), 3.47 (s, 3H), 3.56-3.50 (m, 3H), 3.78-3.68 (m, 1H), 4.32 (d, 1H, J = 10.0 Hz), 4.53 (brs, 1H), 4.95 (brs, 2H), 5.20 (s, 1H), 5.86 (t, 1H, J = 10.6 Hz), 5.94 (d, 1H, J = 9.4 Hz), 6.60 (t, 1H, J=11.4), 6.97 (d, 1H, J = 11.6 Hz), 7.08 (br s, 1H), 7.26 (s, 1H), 9.21 (s, 1H).

**Compound 5**: 17-[2-(Piperazin-1-yl)ethyl]amino-17-demethoxygeldanamycin was synthesized following Method A and was obtained as a purple solid from geldanamycin and 2-(1-piperazinyl)ethylamine, with a yield of 49%. ^1^H NMR(400 MHz, MeOD): δ=0.80 (d, 3H, J = 6.48 Hz), 0.99 (d, 3H, J = 7.2 Hz), 1.61–1.76 (m, 3H), 1.76 (s, 3H), 2.02 (s, 3H), 2.36 (m, 1H), 2.46 (m, 4H), 2.56 (m, 2H), 2.64–2.77 (m, 2H), 2.96 (t, 4H, J = 4.56 Hz), 3.30 (s, 3H), 3.36 (s, 3H), 3.48–3.53 (m, 2H), 3.56 (m, 1H), 3.68 (m, 1H), 4.55 (d, 1H, J = 8.56 Hz), 4.95 (br s, 2H), 5.23 (s, 1H), 5.37 (d, 1H, J = 10.16 Hz), 5.63 (t, 1H, J = 10.56 Hz), 5.88(t, 1H, J = 9.28 Hz), 6.66 (br s, 1H), 7.08 (s, 1H), 7.15 (d, 1H, J = 11.9 Hz), 9.21 (s, 1H).

**Compound 6**: 17-[2-(Piperidin-4-yl)ethyl]amino-17-demethoxygeldanamycin was synthesized following Method A and was obtained as a purple solid from geldanamycin and 4-piperidineethanamine, with a yield of 33%. ^1^H NMR(400 MHz, MeOD): *δ*= 1.00 (d, 3H, J = 5.4 Hz), 1.12 (t, 2H, J = 6.3 Hz), 1.16 (d, 3H, J = 7.3 Hz), 1.27 (t, 2H, J = 7.3 Hz), 1.30-1.45 (m, 1H), 1.57–1.69 (m, 3H), 1.75 (s, 3H), 2.02 (s, 3 H), 2.30 (m, 1H), 2.75 (m, 2H), 3.19 (d, 2H, J = 7.3 Hz), 3.25 (m, 4H), 3.33 (s, 3H), 3.38 (s, 3H), 3.42–3.50 (m, 2H), 3.53 (m, 1H), 3.62 (m, 1H), 4.55 (d, 1H, J = 8.3 Hz), 4.94 (br s, 2H), 5.23 (s, 1H), 5.89 (t, 1H, J = 8.9 Hz), 5.91 (d, 1H, J = 9.6 Hz), 6.64 (t, 1H, J = 11.9 Hz), 7.06 (br s, 1H), 7.14 (d, 1H, J = 11.6 Hz), 7.92 (s, 1H), 9.18 (s, 1H).

**Compound 7**: 17-[4-(Guanidin-1-yl)butyl]amino-17-demethoxygeldanamycin was synthesized following Method B and was obtained as a purple solid from geldanamycin and N-(4-aminobutyl)guanidine, with a yield of 35 %. ^1^H NMR (400 MHz, MeOD): *δ*= 1.00 (d, 3H, J = 6.9 Hz), 1.02 (d, 3H, J = 6.8 Hz), 1.54-1.63 (m, 4H), 1.64-1.68 (m, 1H), 1.69-1.80 (m, 8H), 1.81-1.90 (m, 2H), 2.03 (s, 3H), 2.25-2.35 (m, 2H), 2.68-2.79 (m, 3H), 2.89 (s, 3H), 3.02 (s, 3H), 3.14-3.17 (m, 1H), 3.45-3.52 (m, 2H), 3.60-3.66 (m, 2H), 3.69-3.83 (m, 3H), 4.56 (d, 1H, J = 8.2), 5.25 (s, 1H), 5.61 (d, 1H, J = 10.4 Hz), 5.90 (d, 1H, J = 10.4 Hz), 6.64 (t, 1H, J=11.7 Hz), 7.08 (br s, 1H), 7.16 (d, 1H, J = 11.4 Hz).
